# Predictors of receiving therapy among very low birth weight 2-year olds eligible for Part C early intervention in Wisconsin

**DOI:** 10.1186/1471-2431-13-106

**Published:** 2013-07-11

**Authors:** Beth Marie McManus, Stephanie Robert, Aggie Albanese, Mona Sadek-Badawi, Mari Palta

**Affiliations:** 1 Department of Health Systems, Management & Policy, Colorado School of Public Health, 13001 E. 17th Place, MS B117, Aurora, CO 80045, USA; 2 Department of Social Work, University of Wisconsin-Madison, Madison, WI, USA; 3 Department of Population Health Sciences, University of Wisconsin-Madison, Madison, WI, USA

**Keywords:** Very low birth weight, Early intervention, Physical therapy, Neighborhood disadvantage

## Abstract

**Background:**

The Individuals with Disabilities Education Act (Part C) authorizes states to establish systems to provide early intervention services (e.g., therapy) for children at risk, with the incentive of federal financial support. This study examines family and neighborhood characteristics associated with currently utilizing physical, occupational, or speech therapy among very low birthweight (VLBW) 2-year-old children who meet Wisconsin eligibility requirements for early intervention services (EI) due to developmental delay.

**Methods:**

This cross-sectional analysis used data from the Newborn Lung Project, a regional cohort study of VLBW infants hospitalized in Wisconsin’s newborn intensive care units during 2003–2004. We included the 176 children who were age two at follow-up, and met Wisconsin state eligibility requirements for EI based on developmental delay. Exact logistic regression was used to describe child and neighborhood socio-demographic correlates of parent-reported receipt of therapy.

**Results:**

Among VLBW children with developmental delay, currently utilizing therapy was higher among children with Medicaid (aOR = 5.3, 95% CI: 1.3, 28.3) and concomitant developmental disability (aOR = 5.2, 95% CI: 2.1, 13.3) and lower for those living in a socially more disadvantaged neighborhood (aOR=0.48, 95% CI: 0.21, 0.98, per tertile).

**Conclusions:**

Among a sample of VLBW 2-year olds with developmental delays who are EI-eligible in WI, 4 out of 5 were currently receiving therapy, per parent report. Participation in Medicaid positively influences therapy utilization. Children with developmental difficulties who live in socially disadvantaged neighborhoods are at highest risk for not receiving therapy.

## Background

Children born very low birth weight (<1,500 grams, VLBW) are at heightened risk for significant developmental difficulties [1,2]. To mitigate disability associated with very low birth weight, the World Health Organization and the March of Dimes [3] advocate for bolstering infant and child health and education initiatives (e.g., early intervention) for all children born very low birth weight.

In the U.S, the Individuals with Disabilities Education Act (IDEA), Part C, authorizes states to establish systems to provide early intervention (EI) services for children with developmental delays and disabilities, with the incentive of federal financial support [4]. EI is not a single program, but rather includes different packages of services that vary within and across states with regard to delivery models and provider disciplines [5]. Due to the flexibility of EI service delivery models, measuring which children receive what type of EI services is difficult. To address this methodologic challenge, we focus our study on a narrow aspect of EI service delivery –physical, occupational, or speech therapy. Indeed, data from the National Early Intervention Longitudinal Study (NEILS), an observational cohort of children enrolled in over 20 EI programs across the United States, suggests that 14% of parents report wanting more physical, occupational, or speech therapy services for their children [6]. This suggests that research focusing on predictors of therapy utilization among EI-eligible children is justified to better understand, in a sample of infants at high risk for poor developmental outcomes, what proportion receives therapy services. Moreover, some studies suggest that therapy services may improve cognitive and motor skills among very low birth weight infants [7,8]. Thus, understanding predictors of therapy services among this vulnerable population has important clinical and programmatic implications. For example, given the recent national evidence suggesting overall difficulties in access to EI [9,10], shedding light on predictors of a specific aspect of EI services (i.e., therapy) seems critical to improving our understanding of access disparities.

We focus our inquiry on specific therapy services among a sample of VLBW infants eligible for EI in Wisconsin. Our assumption is that EI is the most common source of physical, occupational, and speech therapy services. Thus, our results about therapy services may translate to predictors of EI therapy since we restrict our inquiry to an EI-eligible population. However, the extent to which our results capture therapy services received outside of EI (e.g., clinic-based therapies) among this high-risk, EI-eligible sample might suggest barriers to accessing EI as these vulnerable young children should be (i.e., according to IDEA mandates) receiving EI-based versus clinic-based therapy.

As summarized briefly below, health insurance, race, and socioeconomic status have all been shown to be associated with use of developmental and therapeutic services. Although there is some evidence about the role of neighborhood socioeconomic context as a predictor of use of developmental and therapeutic services, there has been less attention to this potentially important contextual factor.

Comprehensive health insurance for low-income children is provided through state Medicaid programs [11]. Children’s Medicaid mandates Early and Periodic Screening, Diagnosis, and Treatment (EPSDT) to meet the unique physical, emotional, and developmental needs of low income children [12]. The role of Medicaid in facilitating access to therapy services for very young children is not clear. While some previous studies suggest children enrolled in Medicaid have fewer unmet therapy needs [13,14] than those with private insurance, other studies suggest Medicaid enrolled children are less likely to access EI [10,15].

In theory, insurance status (Medicaid participation versus having private insurance) should not be expected to influence therapy utilization for infants and toddlers eligible for early intervention since eligibility for early intervention is not contingent on health insurance access. However, in recent years, financing mechanisms [16] for early intervention have evolved to include more third-party billing and rising family fees, which are waived for children enrolled in Medicaid. Given the current financial crisis of early intervention programming [17] understanding the influence of source of insurance on therapy utilization among children eligible for early intervention has important implications for potentially improving efficiency of early intervention service delivery.

Research consistently shows that poor and minority children are at greater risk for developmental difficulties. For example, EI enrollment data from the National Longitudinal Study of Early Intervention [18] suggest that poor and minority children are over-represented in EI compared to the general population of infants and toddlers. Moreover, studies on EI utilization indicate that poor and minority children also have higher levels of unmet need for EI services than their non-poor and non-minority counterparts [10,15,19,20].

Neighborhood socioeconomic conditions have been associated with a range of health, health care, and access outcomes among children [21]. Less is known about whether neighborhood socioeconomic context (i.e., neighborhood social disadvantage) may contribute to access to early intervention services. Some research demonstrated that neighborhood poverty is associated with longer time from referral to initiation of early intervention services [22]. But is neighborhood socioeconomic context associated with utilization of any intervention services? Neighborhood socioeconomic context (i.e., neighborhood social disadvantage) may affect access to early intervention services through characteristics of both the service environment (e.g., fewer services, poor transportation options) and the social environment (e.g., social norms, information networks).

To generate understanding of factors that affect utilization of early intervention therapy services, we take advantage of an existing regional cohort to study an early intervention-eligible subgroup (i.e., VLBW children with developmental delay) of a statewide cohort of VLBW children in Wisconsin. We hypothesize that, among two-year-olds born VLBW and eligible for early intervention therapy services due to developmental delay, children not currently receiving therapy will be more likely to be living in neighborhoods characterized by social disadvantage (i.e. an indicator for lack of adequate health and developmental resources).

Understanding social disparities in utilization of therapy services among VLBW children eligible for early intervention has important policy and programmatic implications for improving outreach and therapeutic service delivery to high-risk families.

## Methods

### Study population

The Newborn Lung Project is a regional cohort of infants born VLBW in 2003–2004 and hospitalized in any of the 16 Newborn Intensive Care Units in Wisconsin. The principal investigator of the Newborn Lung Project (MP) granted permission to the first author (BMM) to access and analyze the data. The original cohort recruited 979 infants for whom de-identified clinical data and follow-up contact information were collected. At age 2–3, 719 children had parent questionnaire and child developmental assessment data collected. In the present analysis, data for 26 Spanish-only speaking families were omitted because the developmental assessment was not available in Spanish. Of the 693 remaining children, 191 were age 2 and met Wisconsin early intervention eligibility criteria (i.e., performance more than 1.5 standard deviations below the mean on a developmental assessment) [23]. The 191 included only 6 Hispanic and 5 Asian, Hawaiian, or multi-racial children. Due to these small numbers, we restrict our analyses to white and black, non-Hispanic children (n=176).

Developmental performance was measured using the Pediatric Evaluation of Disability Index (PEDI) [24]. The PEDI is a functional assessment appropriate for children ages 6 months to 7 years. Social, motor, and self-care items are scored dichotomously by parents based upon the child’s ability to perform the task in most situations (1) or unable to perform the task in most situations (0). Raw scores are converted to a normalized, age standardized T-score (mean=50, SD=10). Children were included in the sample analyzed here if they had a PEDI score (in motor, self-care, or social domain) of <35. According to Wisconsin mandates for early intervention, the PEDI is an accepted tool to determine eligibility for early intervention [25].

Mean PEDI scores, by therapy status (receiving therapy vs. not), are presented in Table 1.

**Table 1:** **Descriptive statistics of receiving therapy among a sample (n=176) 2 year olds born very low birth weight and eligible for early intervention in Wisconsin**^A^

	**Therapy (n=142)**	**No therapy (n=34)**
Child’s race		
White, non-Hispanic	112 (79.4)	29 (20.6)
Black, non-Hispanic	30 (85.7)	5 (14.3)
Total Annual Income		
Less than $30,000	57 (89.1)	7 (10.9)
$30,000 - $60,000	41 (80.4)	10 (19.6)
More than $60,000	44 (72.1)	17 (27.9)
Maternal Education		
HS Diploma /Equivalent or Less	45 (81.8)	10 (18.2)
Some post HS schooling	53 (81.5)	12 (18.5)
Bachelor Degree or more	44 (78.6)	12 (21.4)
Sex of the Child		
Boys	79 (79.0)	21 (21.0)
Girls	63 (82.9)	13 (17.1)
Family Structure		
Single-parent household	45 (88.2)	6 (11.8)
Lives with both parents	97 (77.6)	28 (22.4)
Mom received prenatal care		
Yes	139 (81.3)	32 (18.7)
No	3 (60.0)	2 (40.0)
Health Insurance		
Private	53 (37.3)	25 (73.5)
Medicaid	89 (90.8)	9 (9.2)
Developmental status		
Developmental delay only	33 (73.3)	12 (26.7)
Concomitant developmental disability	109 (83.2)	22 (16.8)
Neighborhood disadvantage^B^		
Disadvantaged	44 (81.5)	10 (18.5)
Moderately Disadvantaged	62 (79.5)	16 (20.5)
Advantaged	36 (81.8)	8 (18.2)
		**Mean (SD)**
Child’s Age (chronological, in moths)	28.4 (2.5)	28.4 (3.0)
Mother’s age (years)	31.7 (7.0)	33.3 (5.8)
Birth weight (grams)	939 (272)	1127 (219)
Severity of neonatal morbidity^C^	21.2 (15.5)	13.6 (11.9)
Functional Skills^D^		
Social function	32.8 (12.0)	39.0 (12.6)
Motor function	30.1 (13.0)	37.8 (8.4)
Self-Care	34.2 (10.3)	37.8 (8.4)

^A^ Wisconsin state eligibility criteria for early intervention due to developmental delay is determined by performance on a standardized developmental evaluation that is more than 1.5 standard deviations below the mean.

^B^ Neighborhood disadvantage categories were created from an overall neighborhood disadvantage index (combining maternal education, poverty, single-family households, maternal unemployment, and incomes below state median each collected at the Census tract level; higher scores indicate more disadvantage) to correspond to disadvantaged (highest tertile), moderate disadvantaged (middle tertile), and advantaged (lowest tertile).

^C^ Severity of Neonatal morbidity is measured using the Score of Neonatal Acute Physiology (SNAP-II, range 0–115). Higher scores indicate more severe morbidity.

^D^ Functional skills were measured by the Pediatric Evaluation of Disability Inventory (PEDI). The PEDI is a norm-referenced developmental assessment tool with a mean of 50 and standard deviation of 10. The PEDI meets Wisconsin mandates for a developmental tool appropriate for determining eligibility for EI services.

### Outcome variable

The primary outcome of interest, receipt of therapy, was collected by parent-report of whether the child currently receives physical, occupational, or speech therapy services (any services vs. no services).

### Child and family characteristics

Parental education was categorized as high school (HS) (i.e., 12 years) or less, some college, and completion of a college degree (i.e., at least 16 years). Child’s race and ethnicity was grouped as white, non-Hispanic or black, non-Hispanic (hereafter referred to as black). Annual family income (USA$) was categorized as less than $30,000, $30,000 to $60,000, and greater than $60,000. We also included birthweight, sex of the child, child’s age, mother’s age at child’s birth, whether the child received Medicaid or private insurance, and family structure (single-parent versus two parent families). Severity of neonatal morbidity was measured using the Score for Neonatal Acute Physiology (SNAP) [26], an index ranging from 0–115 that comprises 6 physiologically-based items (pH, urine output, blood pressure, temperature, oxygen requirement, and seizures) with higher scores reflecting more severe morbidity. Child’s developmental status was categorized as developmental delay only or concomitant developmental disability (e.g., cerebral palsy or other neurological conditions). Descriptive statistics of these variables, by therapy status, are presented in Table 1.

### Neighborhood disadvantage

We conceptualized neighborhood disadvantage as social characteristics of neighborhoods that were associated with disadvantage (i.e., low income, low maternal education, single family households, etc.) and because these factors have particular relevance for child development and family well-being. A neighborhood disadvantage index was created using principal component analysis (PCA) of 5 Census tract socio-demographic variables (U.S. Census Bureau, 2003): % families in poverty, % of households with income above state median, % females with bachelor’s degree or more, % single mothers, and % of mothers of young children unemployed. PCA [27] is a data reduction technique that determines how to combine variables into a single score that captures as much as possible of the overall variability in all the variables and has been previously used in perinatal epidemiologic research [28]. Specifically, the 5 Census variables were first standardized (after reverse coding % females with bachelor’s degree or more and % of households with income above state median). An overall neighborhood disadvantage score (mean=0, SD=1, and alpha=0.86) was created as an average of these items weighted by the item loadings (whose elements measure the strength of the relationship between the variable and principal component). The linear index was then split into tertiles, and children were classified as living in either disadvantaged (highest third), moderately disadvantaged (middle third), or advantaged (lowest third) neighborhoods. The results of the PCA have been previously published [29]. Item loadings ranged from 0.66 for unemployed mothers of young children to 0.90 for families living in poverty.

### Analytic approach

To test our study hypotheses, we fit a series of exact logistic regression models. Exact logistic regression is an appropriate method to obtain estimates and confidence intervals with small sub-group sample sizes.

First, we tested for significant bi-variate associations between each child and family characteristic and receipt of therapy services. Significant predictors (*p* <.05) were included in subsequent model building. In the bi-variate analyses, only family income, insurance type, developmental status, and neighborhood disadvantage were statistically significant. We describe the subsequent model building process including these covariates.

The first model included family income to describe its association with current receipt of therapy. The second model additionally included child’s developmental status (i.e., presence of a developmental disability versus developmental delay only), and Medicaid participation to describe their association, above and beyond family income. In the third model we added neighborhood disadvantage to examine its association after controlling for child and family attributes. We modeled neighborhood disadvantage as an ordinal variable. In the multivariable models we present, the interpretation of the neighborhood disadvantage covariate is the difference in odds of receiving therapy between children from a more socially disadvantaged neighborhood category to those from the preceding less socially disadvantaged one (e.g., disadvantaged versus moderate disadvantaged).

For each covariate, we report the odds ratio (and 95% confidence interval) of receiving therapy. All analyses were conducted in SAS v.9.2 [30]. The institutional review board at the University of Wisconsin-Madison and all participating centers (St. Joseph’s Hospital, Milwaukee, WI; Aurora Sinai, Milwaukee, WI; St. Joseph’s, Marshfield, WI Meriter Hospital, Madison, WI; Children’s Hospital of Wisconsin, Milwaukee, WI; St. Mary’s Hospital, Madison, WI; St. Vincent’s Hospital, Green Bay, WI; Columbia-St. Mary’s Hospital, Milwaukee, WI; St. Luke’s Hospital, Racine, WI; Children’s Hospital of Wisconsin – Fox Valley, Neenah, WI; Waukesa Memorial Hospital, Waukesha, WI; Aurora Women’s Pavilion, West Allis, WI; Gunderson Lutheran Hospital, LaCrosse, WI; Aurora Bay Care, Green Bay, WI; St. Elizabeth’s Hospital, Affinity Health System, Appleton, WI; and Franciscan Skemp Hospital, LaCrosse, WI) approved this study. Parents of the study children provided written informed consent to participate.

## Results

Of the full sample (n=176), 142 children (80.7%) had parent-reported current receipt of therapy (Table 1). In Table 2, Model 1 indicates that children from lowest-income families (aOR=3.12 95% CI: 1.1, 9.7) were more likely to currently receive therapy than their non-poor counterparts.

**Table 2:** Unadjusted and adjusted odds ratios (95% confidence interval) of receiving therapy for a sample of very low birth weight children (n=176), who, at age 2, meet Wisconsin state eligibility criteria^A^ for early intervention due to developmental delay

	**Unadjusted OR (95% CI)**	**Adjusted OR (95% CI)**		
		**Model 1**	**Model 2**	**Model 3**
		**Socio-demographic**	**Medical and health service**	**Neighborhood disadvantage**
Family Income				
< $30,000	3.15 (1.20, 8.25)	3.12 (1.11, 9.71)	1.06 (0.18, 5.70)	1.58 (0.22, 11.57)
$30,000 - $60,000	1.99 (0.70, 5.65)	1.58 (0.60, 4.34)	1.13 (0.37, 3.61)	1.42 (0.43, 4.96)
>$60,000	Reference	Reference	Reference	Reference^C^
Developmental status				
Developmental delay	Reference		Reference	Reference
Developmental disability	6.06 (2.71, 13.53)		4.84 (1.95, 12.52)	5.15 (2.1, 13.27)
Health Insurance				
Private	Reference		Reference	Reference
Medicaid	4.66 (2.03, 10.74)		3.80 (0.92, 19.48)	5.26 (1.25, 28.33)
Neighborhood	1.01 (0.61, 1.67)			0.48 (0.21, 0.98)
Disadvantage^B^				

^A^ Wisconsin state eligibility criteria for early intervention due to developmental delay is determined by performance on a standardized developmental evaluation that is more than 1.5 standard deviations from the mean.

^B^ Neighborhood disadvantage categories were created from an overall neighborhood disadvantage index (combining maternal education, poverty, single-family households, maternal unemployment, and incomes below state median each collected at the Census tract level; higher scores indicate more disadvantage) to correspond to disadvantaged (highest tertile), moderate disadvantaged (middle tertile), and advantaged (lowest tertile) and modeled as an ordinal variable. The odds ratio can be interpreted as the difference in odds of receiving therapy between children from one neighborhood category to those from the preceding less disadvantaged one (e.g., disadvantaged versus moderate disadvantaged).

^C^ Reference refers to reference group.

The results of Model 2 (Table 2) suggest that children with concomitant developmental disability (aOR=4.84, 95% CI: 2.0, 12.5) have increased odds of currently receiving therapy compared to VLBW children with developmental delay only. Moreover, adding disability status in Model 2 reduces the magnitude of the parameter estimate of being in the lowest income stratum from aOR=3.12 (Model 1) to aOR=1.06 (Model 2). This suggests that the lowest income children are also likely to have a concomitant disability, which explains (in part) their higher odds of receiving therapy services.

The results of Model 3 demonstrate that children living in a more socioeconomically disadvantaged neighborhood have lower odds of receiving therapy. Specifically, children living in more socioeconomically disadvantaged neighborhoods had 52% lower odds (aOR=0.48, 95% CI: 0.21, 0.98) of currently receiving therapy than their peers living in advantaged neighborhoods, after controlling for family socio-demographic characteristics and severity of child’s condition. Also of note, after controlling for children’s family and neighborhood socioeconomic characteristics, children participating in Medicaid have significantly higher odds (aOR=5.26, 95% CI: 1.25, 28.33) of receiving therapy than their privately insured counterparts.

**Figure 1:**
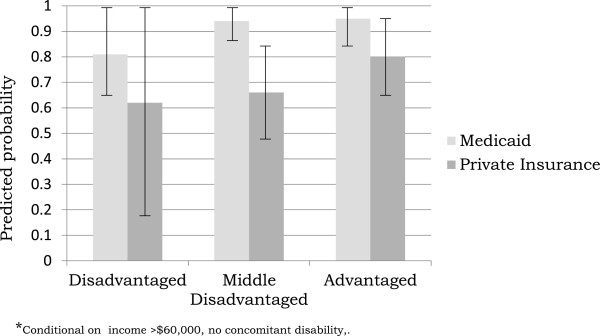
Adjusted predicted probability (and 95% CI) of receiving therapy by neighborhood social disadvantage category and insurance type among a sample (n=176) of VLBW 2 year olds with development delay.

In sum, the results suggest that children with the highest odds of receiving therapy services are insured through Medicaid and do not live in a socially disadvantaged neighborhood. To further explain these findings, we conducted post hoc analyses. A final model (post hoc) additionally included an interaction term between neighborhood social disadvantage and insurance type. This model tested the differential effect, on receipt of therapy services, of having Medicaid for children living in neighborhoods with different levels of social disadvantage. To assist with interpretation of the interaction term, we modeled neighborhood disadvantage as a categorical variable and present the adjusted predicted probability of receiving therapy services, by insurance type, for each category of neighborhood social disadvantage. In the final model, the interaction term was statistically significant (p<.05) suggesting that the influence of Medicaid on receipt of therapy services *differs* for different levels of neighborhood social disadvantage. Moreover, for children living in more disadvantaged neighborhoods, having Medicaid seems particularly helpful for utilizing therapy services (Figure 1).

## Discussion

Among an existing regional cohort of VLBW 2-year olds, we examined family and neighborhood correlates of current receipt of physical, occupational, or speech therapy among a sample with developmental delay who meet state eligibility criteria for receipt of early intervention services. Among this high-risk population eligible for EI services, overall receipt of therapy is high (80%) and children with concomitant developmental disability are more likely to receive therapy. Moreover, we found that children with Medicaid had higher odds of receiving therapy than their privately insured peers and children living in a more socioeconomically disadvantaged neighborhood had lower odds of accessing any therapy services.

Our results regarding Medicaid use are consistent with a previous study from another state (i.e., Massachusetts) [13] suggesting that children with developmental risk enrolled in Medicaid have fewer unmet therapy needs. However, our findings differ from nationally representative samples [14] of children with developmental difficulties in which Medicaid enrollment did not influence receipt of services. This suggests that the impact of Medicaid on service use may vary from state to state. Our results are consistent with other research indicating that Medicaid participation can ameliorate access and navigation difficulties facing low-income families with children with developmental disabilities [31]. Wisconsin and Massachusetts have both led the country in optimizing access to children’s Medicaid programming, which likely explains why Medicaid has a positive association with access to services in both of these states. This provides an optimistic message that high quality Medicaid programming may actually enhance the use of services among at risk children. But why are rates of utilization higher for Medicaid participants than those with private insurance? We consider two reasons. First, annual family participation fees are waived for children insured through Medicaid. Perhaps this removes a financial barrier to accessing early intervention services. Secondly, the EPSDT mandate for Medicaid might encourage pediatricians and family physicians to facilitate an early intervention referral for families. Regardless of the mechanism, as the states move toward restricting eligibility criteria for receipt for early intervention services, it will be important to ensure that children experiencing dual risk – social and developmental – receive necessary therapeutic services. Integrating Medicaid child health and early intervention mandates improve quality (e.g., care coordination) and prevent gaps in service delivery for low-income children living in socially vulnerable neighborhoods. However, we acknowledge that the association we find between Medicaid and therapy utilization could be due to the correlation between Medicaid and a host of child, family, and neighborhood adverse social characteristics (i.e., multicollinearity). Future research with larger sub-sample groups should investigate these associations further.

In the current study, children living in neighborhoods characterized by socioeconomic disadvantage are least likely to receive therapy, even after considering their family characteristics. Within the federal IDEA mandate, early intervention service delivery is largely driven by state and local funding and community advocacy [1]. Thus, it is quite plausible that differences in service delivery are highly correlated with the socioeconomic and advocacy characteristics of communities above and beyond the attributes of their residents. Presumably, more advantaged communities have greater individual and community resource allocation to health and developmental services and higher maternal knowledge and advocacy around child development. Our findings are consistent with a previous study [22] in which children living in poorer neighborhoods were significantly more likely to have delays in receipt of therapy, perhaps due to limited resource allocation. However, we did not directly measure availability of services, only use of service, so future research should investigate how the availability of services, for example, the number of trained pediatric physical, occupational, and speech therapists, influences access to and utilization of therapy services. In addition, parents living in socially disadvantaged neighborhoods may choose not to access therapy services, perhaps due to a cultural stigma of having a child with a developmental delay or disability, or they do not perceive that the child needs or would benefit from therapy services.

Potential mechanisms to explain the association between family and neighborhood sociodemographic risk and unmet need include factors that influence access and adherence to intervention regimens. Previous research [14] suggests that unmet need for therapy services in the U.S. stems from difficulty obtaining referrals for specialty services, finding a skilled provider, and obtaining an adequate number of visits with these providers. Thus, it is plausible that, above and beyond the influence of family income and low maternal education, neighborhood social disadvantage may be an indicator of access and compliance challenges associated with unmet need among this vulnerable population. Indeed, our results suggest an interaction effect whereby the odds of receiving therapy does not differ by insurance type for children living in advantaged neighborhoods. However, for children living in more disadvantaged neighborhoods, insurance type is associated with receipt of therapy services. That is, participation in Medicaid may buffer the ill-effects of living in a socially disadvantaged neighborhood with regard to receipt of therapy services. However, an alternative explanation is that children living in socially disadvantaged neighborhoods who do not have Medicaid are insured through parental employer-sponsored health insurance. For these families, parental work schedules may be a barrier to accessing therapy services. Indeed, our results regarding the intersection between Medicaid participation, neighborhood social disadvantage, and receipt of therapy should be interpreted with the appropriate caution given the small sub-group sample sizes. Future research should explore these potential explanations with larger sample sizes.

We acknowledge several limitations to our study. First, this cross-sectional analysis is limited in its ability to ascribe causality, but rather describes relationships between family and neighborhood attributes and odds of receiving therapy. We acknowledge that parents of children with more therapy needs may be more motivated to apply for Medicaid. Indeed, participation in Medicaid may be a marker for more severe disability (though we did attempt to control for this) as well as maternal motivation and resources, and future research should explore this.

Data collection occurred 8 years ago. As a result, the findings may not reflect current patterns of EI service delivery utilization. However, WI EI eligibility policy has not changed substantially during the last 8 years, which suggests the relevance of the study findings [23,25].

About 1/3 of the original cohort was lost to follow-up. This has the potential to introduce bias if the children who were followed differ from children not followed on characteristics of interest. In unreported sensitivity analyses, we compared analyses with and without propensity score weights derived from measured birth characteristics and found no differences. While this suggests that minimal bias was introduced by differential follow-up, the possibility still exists that children lost to follow up differ on unmeasured variables.

The measurement of neighborhood disadvantage varies across studies. We chose one method (i.e. PCA) that was theoretically driven and methodologically robust to allow inclusion of a broad range of contextual factors while simultaneously addressing their mutlicollinearity. Future research should examine other ways of characterizing neighborhoods and also in understanding the mechanisms linking those neighborhood characteristics to service access.

We were limited in our data collection of therapy utilization to parent-report. We were not able to confirm receipt of therapy services using EI program administrative data (e.g., Individualized Family Service Plans or EI billing data). To our knowledge, the validity of parent report of EI therapy services has not been established. However, previous research [32-35] suggests that parent-report is a valid proxy for administrative health and developmental service utilization data. While these studies did not specifically examine parent-reported EI therapy, we are somewhat reassured that parents accurately report their children’s health and developmental service utilization. Related to this, we asked about current receipt of therapy services. Families responding affirmatively may actually receive clinic-based rather than EI-based services. If this were true, our findings may actually underestimate unmet need for EI therapy as infants and toddlers with developmental delays should be receiving therapy services through Part C EI rather than hospital or outpatient clinics.

Finally, our results may not be generalizable to EI programs in all states. States have flexibility in determining criteria for determining eligibility based upon a developmental delay. Given not only this cut-off (i.e., 1.5 SD below the mean in WI), but also that a parent-reported evaluation tool (i.e., the PEDI) can determine eligibility in WI, differences on these domains may limit applicability of our findings to other states.

## Conclusion

Survival of VLBW children continues to increase with advances in NICU technology, which contributes to increasing numbers of children with developmental delays and disabilities [36]. These life-long consequences for health, development, and quality of life can arguably be ameliorated most effectively with early and continuous intervention. Thus, understanding the types of early intervention service delivery young children with developmental difficulties access has important policy and programmatic implications for improving service delivery and developmental trajectories. Our results suggesting that Medicaid actually facilitates access to services provides hope that there are ways to do so, which should be examined in more detail for replication. However, our results that living in a disadvantaged neighborhood is associated with lower service utilization provides a more sober message that we need to also attend to specific barriers that appear to be occurring in disadvantaged neighborhoods.

## Abbreviations

VLBW: Very low birth weight; EI: Early intervention; PEDI: Pediatric Evaluation of Disability Inventory; SNAP: Score of Neonatal Acute Physiology.

## Competing interests

The authors have no conflicts of interest, financial or otherwise, to disclose.

## Authors’ contributions

BMM conducted the analyses and drafted the manuscript. SAR assisted with drafting the manuscript. AA and MS participated in the design of the study and data collection. MP conceived of the study, and participated in its design and coordination and helped to draft the manuscript. All authors have read and approved the manuscript.

## Pre-publication history

The pre-publication history for this paper can be accessed here:

http://www.biomedcentral.com/1471-2431/13/106/prepub
